# Pozzolanic activity of kaolins containing aluminum hydroxide

**DOI:** 10.1038/s41598-020-70146-3

**Published:** 2020-08-06

**Authors:** Claudia Charlotte Tchamo Leussa, Laurent Libessart, Chafika Djelal, Chantale Njiomou Djangang, Antoine Elimbi

**Affiliations:** 1 Laboratoire de Génie Civil Et géo-Environnement (LGCgE), Université D’Artois ULR 4515, F-62400 Béthune, France; 2 Laboratory of Applied Inorganic Chemistry, University of Yaounde I, POB 812, Yaounde, Cameroon

**Keywords:** Composites, Mechanical properties, Pollution remediation

## Abstract

The addition of 10 wt% aluminum hydroxide to two crude kaolinitic clays, a commercial and a natural freshly mined one, has enhanced their pozzolanic activity, more substantially in the natural sample containing gibbsite. The obtained blends were used as replacement of 20 wt% of Portland cement in the formulations of pastes and mortars which exhibited significant decrease of setting time and increase of compressive strength from early age to 28 days. Also, SEM/EDX analyses showed very heterogeneous structures with hydrated phases identified from XRD. Specific interpretation of the role played by aluminum hydroxide revealed its aptitude to promote the formation of metastable hydrated phases (CAH_10_/C_2_AH_8_) at early age, which temporally inhibited the hydration of cement. This progressive transformation led to the formation of more stable hydrated phases such as C–A–S–H which favored the increase of mechanical strength of the specimens. The sequence of transformation reactions is fully obtained with limited aluminum hydroxide content in clays. Either added as synthetic or naturally occurring in clays, aluminum hydroxide has close role in the strengthening process of cement. Hence, kaolinitic clays that naturally contain gibbsite are suggested as suitable supplementary cementitious material for partial replacement of cement.

## Introduction

Home ownership is fundamental to man’s sense of identity and his commitment to civil society. The use of natural resources to produce low cost construction materials is a cost-effective and sustainable solution to the acute shortage of adequate shelter especially in developing countries where lack of safe, decent and affordable housing is among the greatest impediments to self-fulfillment. In addition to address housing problems, the exploitation of raw minerals is fundamental for socio-economic development. In Cameroon, many studies have identified exploitable kaolinitic clay deposits that are however scarcely used by cement industries^1,2^.

In general, the pozzolanic activity of raw clay materials is limited due to some peculiarities such as particles size, Si/Al molar ratio, lime/pozzolana ratio, water to binder ratio, specific surface area or curing conditions. From chemical and mineral points of view, natural clays contain essentially silica and alumina bounded together by strong chemical bonds that do not easily intervene in reactions such as hydration sequences of the cement phases (C_3_S, C_2_S, C_4_AF and C_3_A) responsible for mechanical strength^3,4^. Heat treatment is among the alternative methods used to efficiently activate clays to a structure where constitutive phases (silica and alumina) can be easily involved in reactions both at fresh and hardened states of mortars and concretes. From previous studies, the range of 500 – 800 °C constitutes the optimum temperatures that transform kaolinitic clay and aluminum hydroxide into amorphous reactive phases^5–13^. Continuous concern to reduce energy consumption in cement industry led to numerous investigations among which are the effect of mechanical activation and the role of some non-clayed phases in raw clays such as gibbsite in the improvement of pozzolanic activity at ambient or lower calcination temperatures^4,8,14^. On this line, the presence of gibbsite in raw kaolinitic clay can lead to high pozzolanic activity and to simultaneous formation of both C–S–H (Calcium Silicate Hydrate) and C–A–S–H (Calcium Aluminum Silicate Hydrate) phases, which promote good mechanical strength. Additionally, the satisfactory pozzolanic activity of amorphous aluminum was reported^15^. From these references, the improvement of the pozzolanicity using aluminum hydroxide to optimize kaolinitic clay properties known already as potential supplementary cementitious materials is feasible. Furthermore, since explanation on the correlation between pozzolanic activity and mechanical strength through these mineral resources is not clarified enough in literature, and/or the use of clays as supplementary cementitious material requires prior activation, all further investigations are beneficial to respond to the societal need of reducing CO_2_ emissions^4,16^. Accordingly, the present study addresses the use of kaolinitic clays in cement production in the context of saving natural resources and reducing CO_2_ emissions. It assesses the effect of amorphous aluminum hydroxide mixed with kaolinitic clays on the strength of pastes/mortars. For this purpose, amorphous aluminum hydroxide was mixed at 10 wt% to two kaolinitic clay materials in order to obtain blends that were used to replace 20 wt% of Portland cement. The pozzolanic activity of the starting kaolinitic clay powders along with their aforementioned mixtures was investigated using the Chapelle test, X-ray Diffraction and Scanning Electron Microscopy to evaluate the formation of hydrated phases. In addition, setting time of pastes was measured as well as compressive strength and strength activity index of mortars.

## Methods

### Materials sampling and characterisation

**Figure 1:**
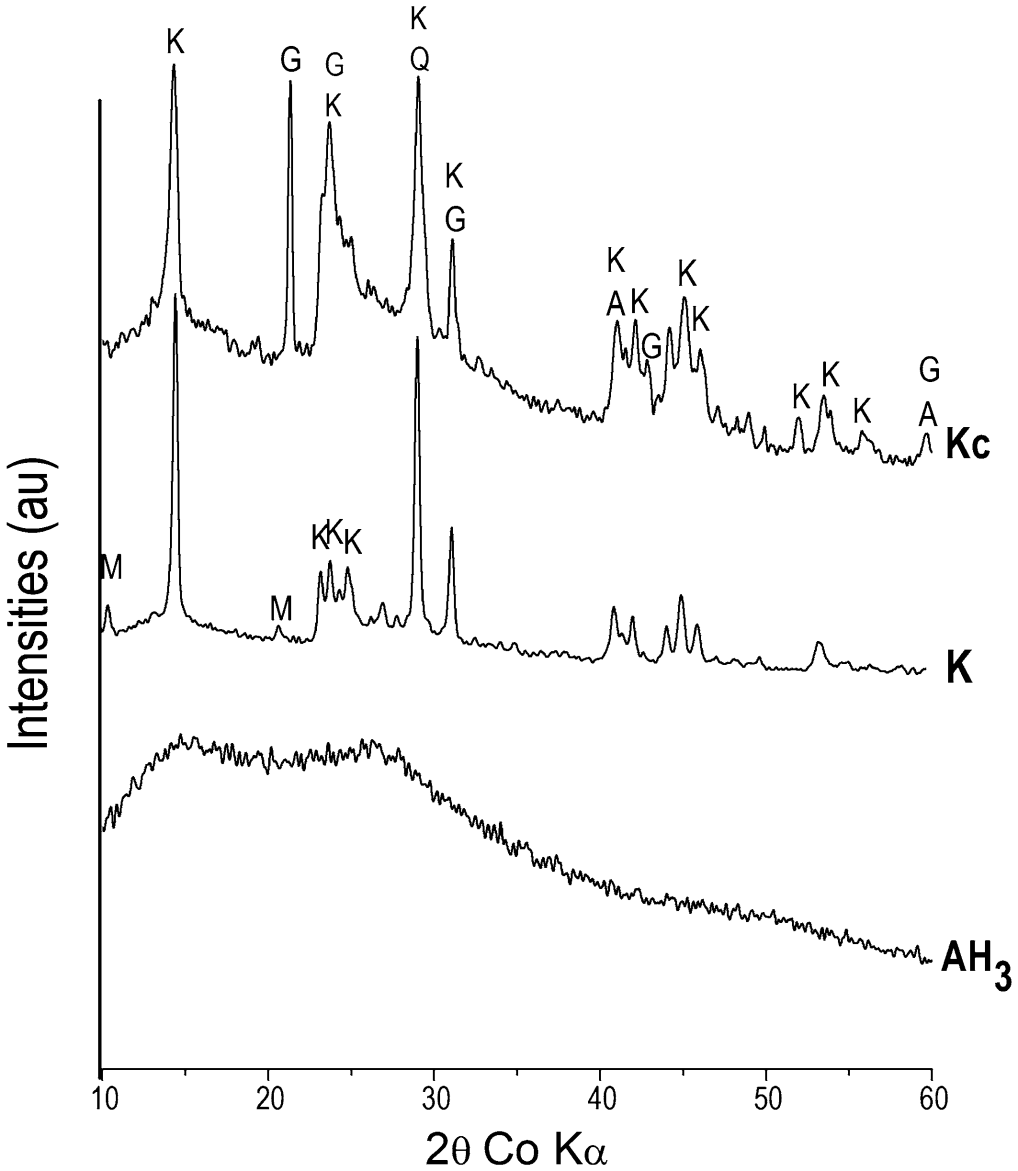
X-Ray Diffraction patterns of starting materials K, Kc and AH_3_; K: Kaolinite; A: Anatase; Q: Quartz; G: Gibbsite; and M: Muscovite.

**Figure 2:**
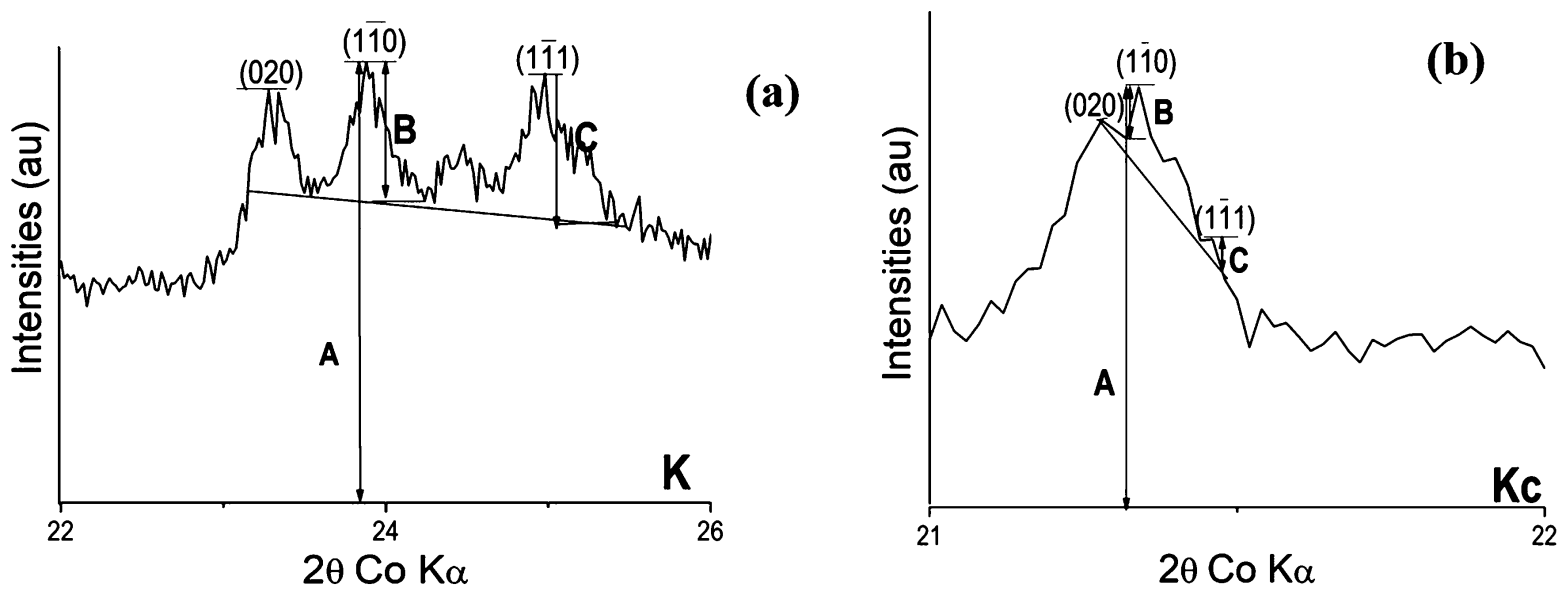
Determination of Hinckley index of (**a**) K, (**b**) Kc.

**Figure 3:**
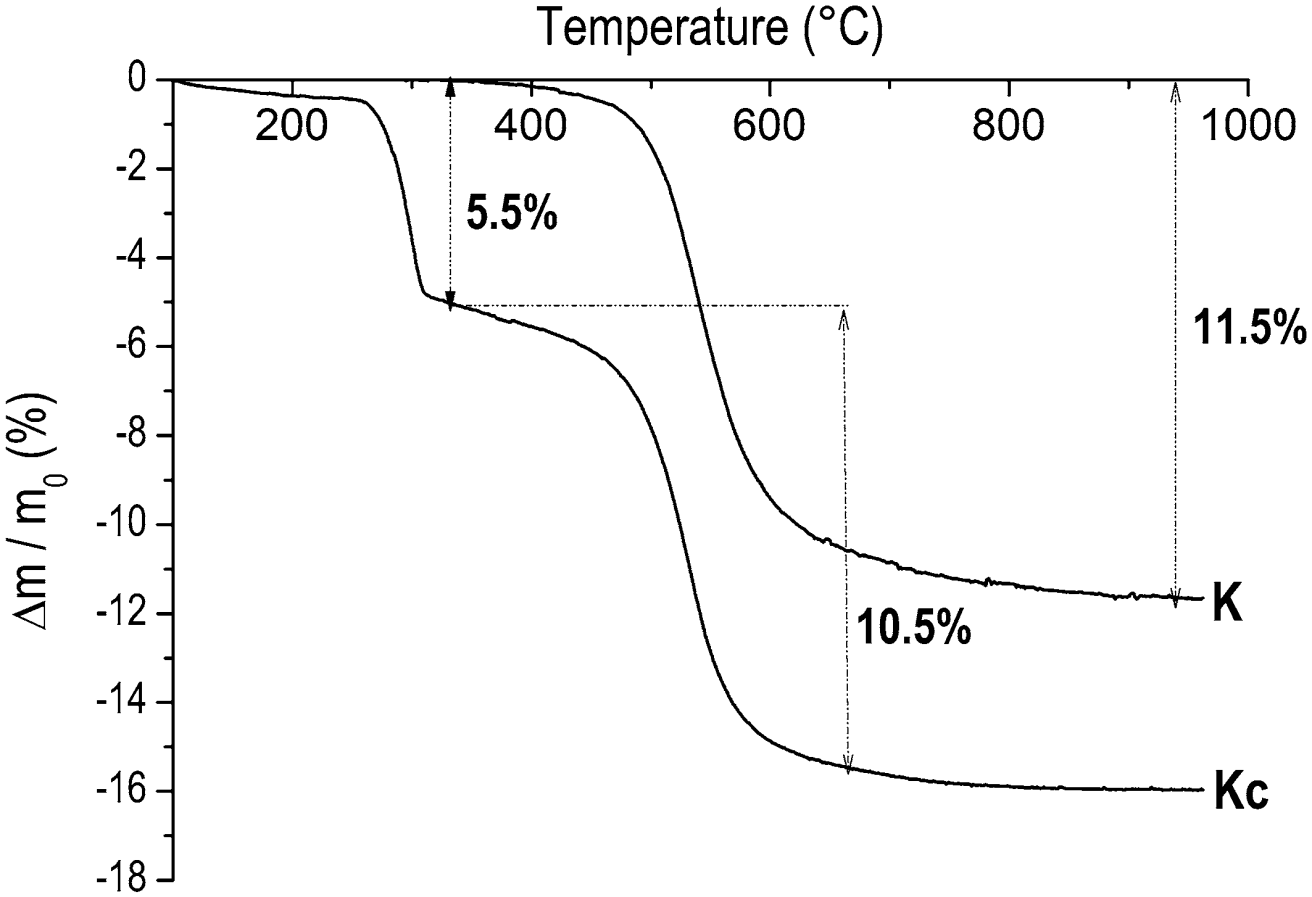
TG curves of K and Kc.

Aluminum hydroxide (AH_3_), from Sigma Aldrich (France), was used in addition to two crude kaolinitic clays, one from CERADEL (France) denoted as K and used as received while the other which was quarried in the South Region of Cameroon was wet-sieved < 20 μm and the residue was dried at 105 °C until constant mass to get powder named as Kc. The specific surface area of K, Kc and AH_3_ was measured using the BET method. Chemical composition of AH_3_ was provided by its supplier, Sigma Aldrich, while those of K and Kc were determined by Inductively Coupled Plasma Optical Emission Spectroscopy using a Perkin Elmer Spectrometer (Optima 7,000 DV ICP-OES). Structural phases were determined by X-Ray Diffraction using a Siemens D 5,000 diffractometer featuring a Bragg–Brentano assembly with a back graphite monochromator with cobalt Kα 1 radiation (~ 1.78 Å). From the diffractograms obtained (Fig. 1), the crystallinity of kaolinite was determined by calculating the Hinckley index which is the ratio of the sum of the heights of the reflexions ($1\overline{1}0$) and ($1\overline{1}1$) measured from the inter-peaks background and the height of the ($1\overline{1}0$) peak measured from the overall background (Fig. 2)^8^. In addition, thermogravimetric analyses (TG) were performed on the two clays using a NETZSCH STA 409 instrument under 75 L min^−1^ air flux with temperature rise of 5 °C min^−1^ (Fig. 3). The semi-quantitative phase composition of the clays was obtained by combining XRD, TG and chemical data^14^. The Portland cement (CEM I 52.5 N CP2) made of 97 wt% of clinker and 3 wt% of gypsum was used, its chemical and mineralogical compositions provided are given in Table 1. Sand from Seine River (France) was also used^17^ and its physical properties (Table 1) were calculated from its granulometric analysis done by dry sieving, using a set of sieves. Those are coefficient of uniformity (Cu) = D_60_/D_10_, coefficient of curvature (Cc) = D_30_^2^/D_10_ × D_60_ (where D_60_, D_30_ and D_10_ are particle diameters at 60%, 30% and 10% of passing respectively)^21^ as well as the fineness modulus and sand equivalent which were respectively determined according to ASTM C33 (1999) and ASTM D2419-95 Standards (1998).

**Table 1:** Chemical, physical and semi-quantitative mineralogical characteristics of starting materials.

Oxides (% wt)	Portland cement CEM I 52.5 N	Commercial Kaolinite (K)	Cameroonian Kaolinite (Kc)	Amorphous aluminum Hydroxide (AH_3_)	Sand
**Chemical composition**					
Al_2_O_3_	3.3	37.1	40.1	98.2	
SiO_2_	20.1	59.0	38.0	–	
K_2_O	3.0	2.5	0.2	–	
Fe_2_O_3_	5.2	1.5	0.7	–	
CaO	64.1	–	0.1	–	
TiO_2_	–	–	1.1	–	
Na_2_O	0.3	–	0.0	1.8	
MgO	0.8	–	0.1	–	
SO_3_	3.0	–	0.0	–	
PF	1.8	13.5	18.8	–	
**Semi-quantitative mineralogical characteristics**					
Kaolinite	–	67.0	75.3	–	
Gibbsite	–	–	15.9	–	
Muscovite	–	21.3	–	–	
C_3_A	8.6	–	–	–	
C_3_S	61.0	–	–	–	
C_4_AF	11.1	–	–	–	
**Physical characteristics**					
Specific surface (m^2^/g)	–	13.5	17.0	51.0	–
Density (g/mL)	3.1	2.5	2.4	2.4	2.6
Fineness modulus (FM)					2.2
Coefficient of uniformity (Cu)					1.9
Coefficient of curvature (Cc)					1.0
Sand equivalent %					97.2
Class index					0/4

Preliminary tests with addition of 10 and 20 wt% of amorphous aluminium hydroxide (AH_3_) in the clays (K and Kc) demonstrated the decrease of mechanical strength for the case of 20 wt%. Thus, 10 wt% of AH_3_ was added to K and Kc to obtain powders labelled as K_10_ and Kc_10_ respectively. The pozzolanic activity of K, Kc, K_10_ and Kc_10_ was assessed by the modified Chapelle test according to NF P 18–513 Standard^18^ which is based on the definition of pozzolanic material as the one capable of binding to portlandite (Ca(OH)_2_) in aqueous solution. For this text, 2 g of CaO were mixed with 1 g of each material (K, Kc, K_10_ and Kc_10_) respectively in 250 mL of distilled CO_2_-free water to form solutions of hydrated lime or portlandite. Each mixture was then stirred at 90 ± 5 °C under reflux for 16 h in a 500 mL balloon flask and then allowed to cool to room temperature before filtrating. From the filtrate, the remaining unreacted portlandite was extracted with saccharose and titrated by a 0.1 N hydrochloric acid solution according to equation (Eq. 1)^18,19^, each value considered being the average of three test results made under the same conditions. On the other hand, the precipitated products of reactions with K, Kc, K_10_ and Kc_10_, denoted as K′, Kc′, K_10_′ and Kc_10_′ respectively, were analyzed by XRD to identify constitutive phases. A blank experiment was carried out similarly without the materials under study for comparison^18^.

1 $$ {\text{CaO}}\,\left( {{\text{lime}}} \right)_{{{\text{aqueous}}}} + 2{\text{HCl}}_{{{\text{aqueous}}}} \to {\text{CaCl}}_{{{\text{aqueous}}}} + {\text{H}}_{2} {\text{O}} $$

### Pastes and mortars formulations

Considering the previous studies which indicated 20 wt% as the optimum value of cement substitution by metakaolin^7,20^, pastes and mortars were designed using cement in which 20 wt% was replaced by K_10_ and Kc_10_ (Table 2). For each paste, the water to binder (W/B) mass ratio was 0.39, the value corresponding to the normal consistency of 0.6 ± 0.1 cm given on the Vicat apparatus and used as required by the EN F 196–3 Standard along with that of the control paste (Pc) (with only cement as binder) was 0.25. These pastes were characterized by the measurement of the setting time using the Vicat apparatus according to EN F 196–3 Standard, both at the normal orientation and at the reverse frame; the measurement was repeated three times to have average of results as retained value. Mortars were obtained by adding 1,350 g of sand to each paste in mass ratio of binder to sand (B/S) of 1/3 as indicated by the EN F 196–1 Standard. The good workability, that is the ease and homogeneity with which the fresh blend of starting materials can be mixed, placed, consolidated and finished according to the EN F 196–1 Standard was obtained after many trials for the determination of the value of W/B mass ratio and 0.55, was finally retained for its satisfactory fulfilment. Then, fresh mortars were shaped into parallelepiped test specimens of 4 × 4 × 16 cm^3^ in a metallic mould then vibrated for 15 s on an electrical vibrating table (M & O. type 202, N° 106) in order to remove entrapped air bubbles before storing under a plastic film in a wardrobe at 100% humidity at 20 °C until 3, 7 or 28 days respectively. Control mortar (Mc) was also designed with only cement as binder for comparison. Compressive strength was measured on test specimens using an electrohydraulic press, type ENERPAC 3R according to NF EN 1,015–11 Standard, the result presented was the average of six tests considered at a particular age. The strength activity index, Ic was calculated according to Eq. (2),

2 $$ I_{c} = \frac{{R_{c} }}{{R_{{c\left( {control} \right)}} }} \times 100 $$

**Table 2:** Pastes and mortars formulations.

Formulations	Cement (%)	Binder/sand (B/S)	Clay mixtures (%)		Water/binder (W/B)
			K_10_	Kc_10_	
**Pastes**					
PC	100	–	–	–	0.25
PK_10_	80	–	20	–	0.39
PKc_10_	80	–	–	20	0.39
**Mortars**					
Mc	100	1/3	–	–	0.55
MK_10_	80	1/3	20	–	0.55
MKc_10_	80	1/3	–	20	0.55

where Rc is the compressive strength of the mortars, and Rc_(control)_ the compressive strength of the control mortar respectively.

X-Ray Diffraction (XRD) was performed on powders of mortar specimens aged 28 days while their broken pieces were etched in resin, polished and coated with the gold film before being analyzed by Scanning Electron Microscopy (SEM/EDX) with JSM 5,900 LV mode apparatus.

## Results and discussion

### Characteristics of starting materials

The chemical and the physical properties of starting materials are given in Table 1 whereas their structural phases are presented in Figs. 1 and 3 respectively. The sums of mass percentage (% Al_2_O_3_ + % SiO_2_ + % Fe_2_O_3_) are 97.6% (K) and 78.8% (Kc), both are greater than 70%, proving that the two clays fit one of the main three conditions for pozzolanic materials along with the strength activity index and the amount of amorphous phase^22^ according to the Standard ASTM C618. Specific surface area (SSA) is 51.0 m^2^/g for AH_3_, 17.0 m^2^/g for Kc and 13.5 m^2^/g for K, which indicated that AH_3_ is the most reactive material, followed by Kc and then K. This behavior is normal for AH_3_ since it is in the amorphous state and has easy solubility enabling the mobility of ionic species^18^. XRD patterns confirmed the amorphous state of AH_3_ displaying a large amorphous halo between 2 theta = 20 and 30°^18,23^. Comparatively, XRD patterns of the two clays showed characteristic peaks of crystalline phases, which are mainly kaolinite and quartz, but also muscovite in K and gibbsite in Kc. The 4 characteristic peaks of kaolinite in K between 2 theta = 20° and 30°, are well shaped proving its good crystallinity whereas in Kc, a small amorphous halo is observed characterising its poor crystallinity^8,20^. Accordingly, the Hinckley index (HI) obtained is 0.8 for K and 0.2 for Kc which confirmed the more crystallinity of kaolinite in K than that in Kc. The crystallinity of the kaolinite in Kc may has been affected by the presence of associated gibbsite which has a peculiarity to decrease the crystallinity of kaolinite according to some authors^8,24^ who described a competitive phenomenon during the geological formation of the two minerals.

Furthermore, the occurrence of gibbsite in Kc is associated with a high amount of Al_2_O_3_ (40.10 wt%). These results are confirmed by TG analyses (Fig. 3), which exhibit the mass loss of 5.5% due to the decomposition of gibbsite around 300–350 °C in Kc and the characteristic mass loss connected to kaolinite between 400 and 700 °C which is 11.5% for K and 10.5% for Kc. From the combination of mass loss values for diverse decompositions and chemical compositions, the semi quantitative mineralogical composition of the two clays was obtained (Table 1).

### Effect of amorphous aluminum hydroxide on the pozzolanic activity of clays

The chemical reaction between portlandite (Ca(OH)_2_) and aluminosilicates in aqueous solution is called pozzolanic reaction. It is always slow and leads to the formation of calcium-silicate-hydrates (C–S–H) and calcium-aluminates/silcates-hydrates (C–A–H/C–A–S–H) which are among the various compounds that possess cementing properties (Eq. 3)^25–27^.

3 $$ {\text{CH}}\,({\text{portlandite}}) + {\text{AS}}\,(aluminosilicate) + {\text{H}}_{2} {\text{O}}\,(water) \to {\text{ C}} - {\text{S}} - {\text{H}} + {\text{C}} - {\text{A}} - {\text{H}}/{\text{C}} - {\text{A}} - {\text{S}} - {\text{H}} $$

The dashes indicate that no particular composition is implies^27^. C–S–H is commonly known as amorphous or poorly crystalline calcium silicate hydrate while calcium aluminates/silicates hydrates are usually of high crystallinity. In this generic reaction, the “aluminosilicate”, is acting as pozzolan and can correspond to kaolinite (2SiO_2_·Al_2_O_3_·2 H_2_O), alumina (Al_2_O_3_), silica (SiO_2_), according to the chemical composition of the starting materials (Table 1), which are kaolinitic clays (K and Kc) and aluminum hydroxide (AH_3_). The possible end-products (Eqs. 3–4) formed with lime can hence be calcium silica hydrates on its amorphous state or on its crystalline phase (tobermorites). The formation of tobermorites is favored by SiO_2_ and heat treatment; they are the main strength-contributing components of the pozzolanic behavior, responsible of both strength and dimensional stability.

4 $$ {\text{Ca}}\left( {{\text{OH}}} \right)_{2} \,(portlandite) + 2{\text{SiO}}_{2} \cdot {\text{Al}}_{2} {\text{O}}_{3} \cdot 2{\text{H}}_{2} {\text{O}}\,(kaolinite) + {\text{H}}_{2} {\text{O}}\,(water) \to {\text{CaO}} - {\text{SiO}}_{2} - {\text{H}}_{2} {\text{O}}\,\left( {{\text{C}} - {\text{S}} - {\text{H}}} \right)/{\text{CaO}} - {\text{Al}}_{2} {\text{O}}_{3} - {\text{SiO}}_{2} - {\text{H}}_{2} {\text{O}}\,\left( {{\text{C}} - {\text{A}} - {\text{S}} - {\text{H}}} \right) $$

5 $$ {\text{Ca}}\left( {{\text{OH}}} \right)_{2} \,(portlandite) + {\text{SiO}}_{2} \,(silica) + {\text{H}}_{2} {\text{O}}\,({\text{water}}) \to {\text{Ca}} - {\text{SiO}}_{2} - {\text{H}}_{2} {\text{O}}\,\left( {{\text{C}} - {\text{S}} - {\text{H}}} \right) $$

**Figure 4:**
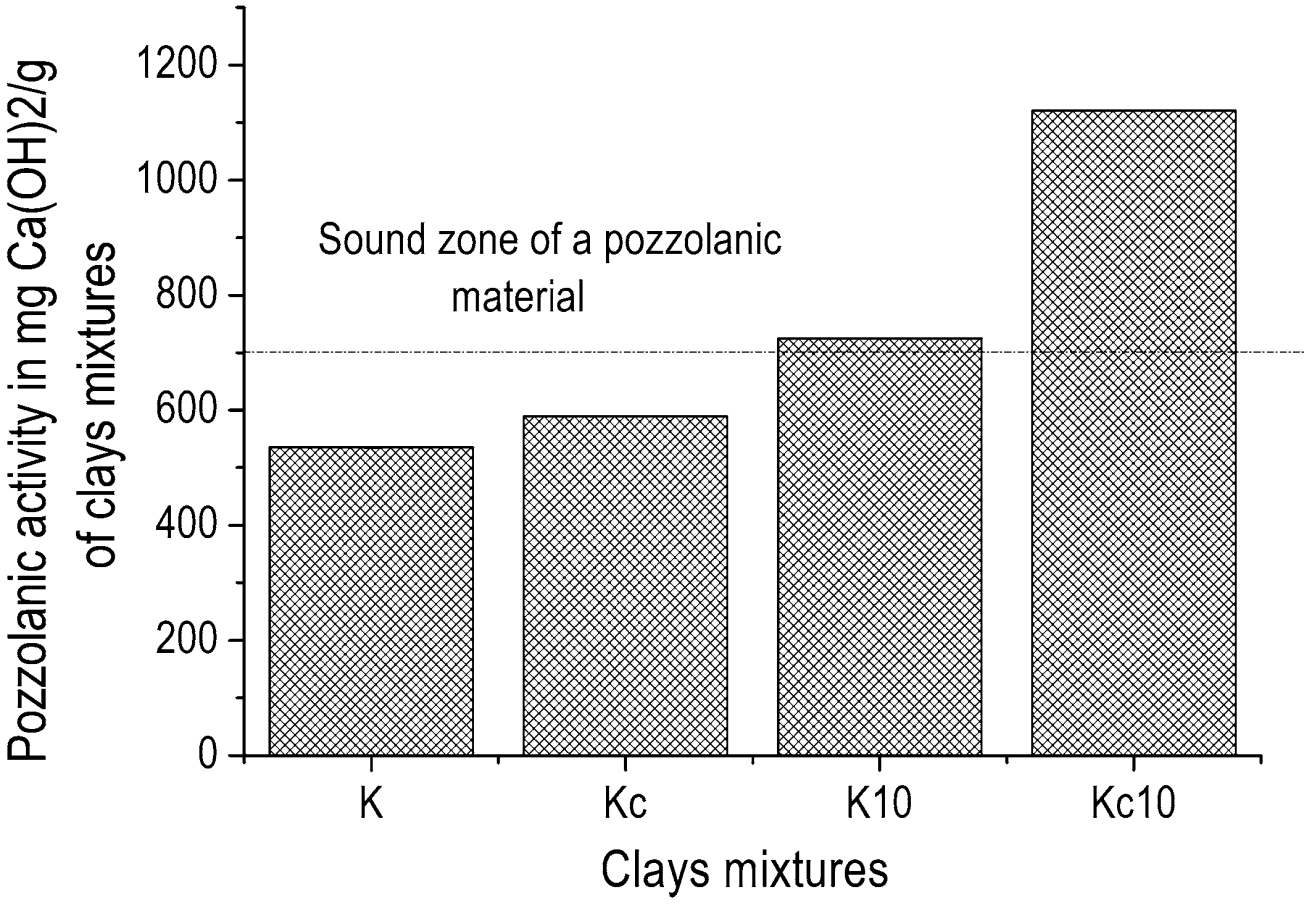
Pozzolanic activity by Chapelle test of clays (K and Kc) and of mixtures clays—10 wt% amorphous aluminum hydroxide (K_10_ and Kc_10_).

**Figure 5:**
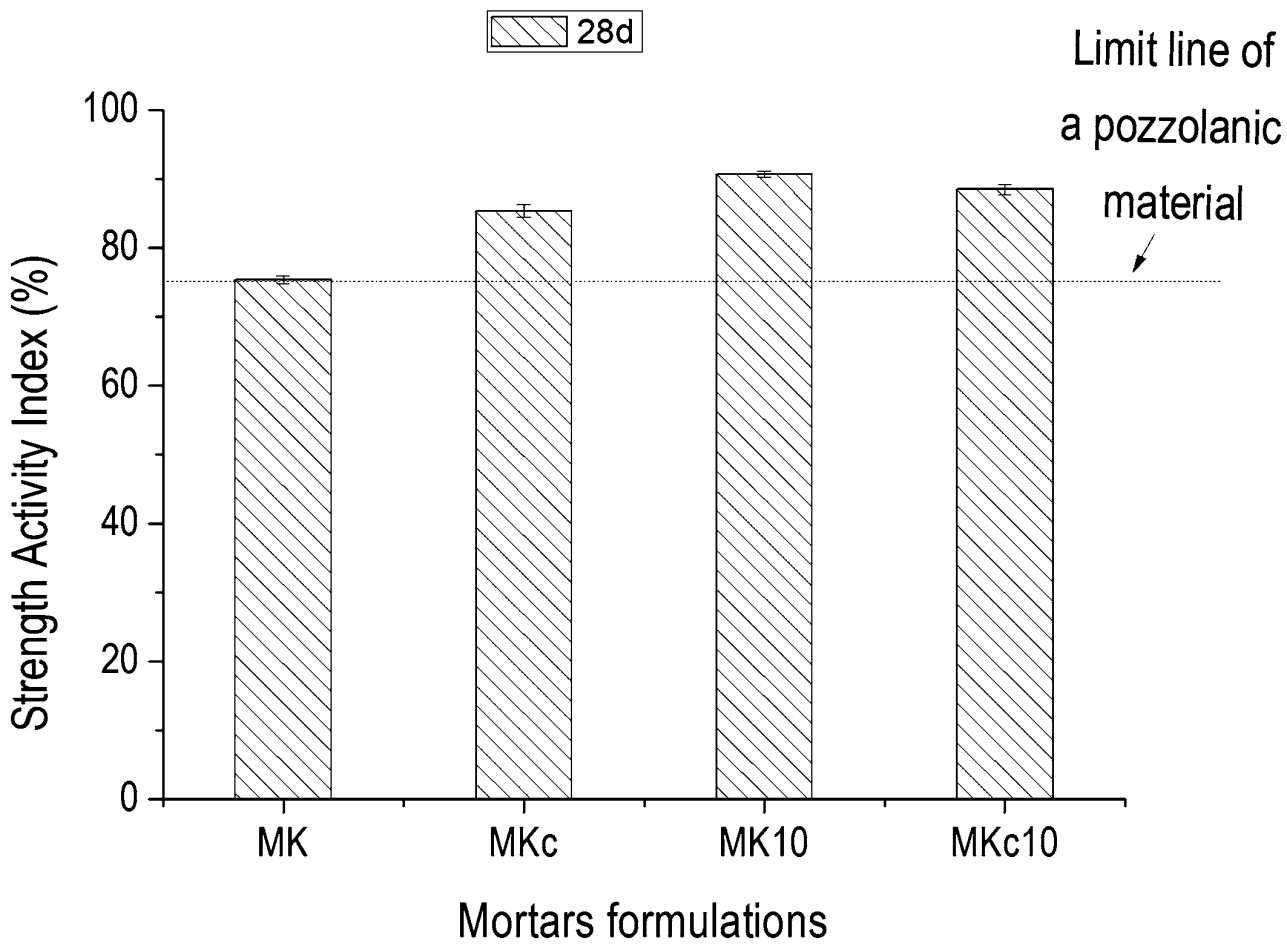
Strength activity index of mortars.

**Figure 6:**
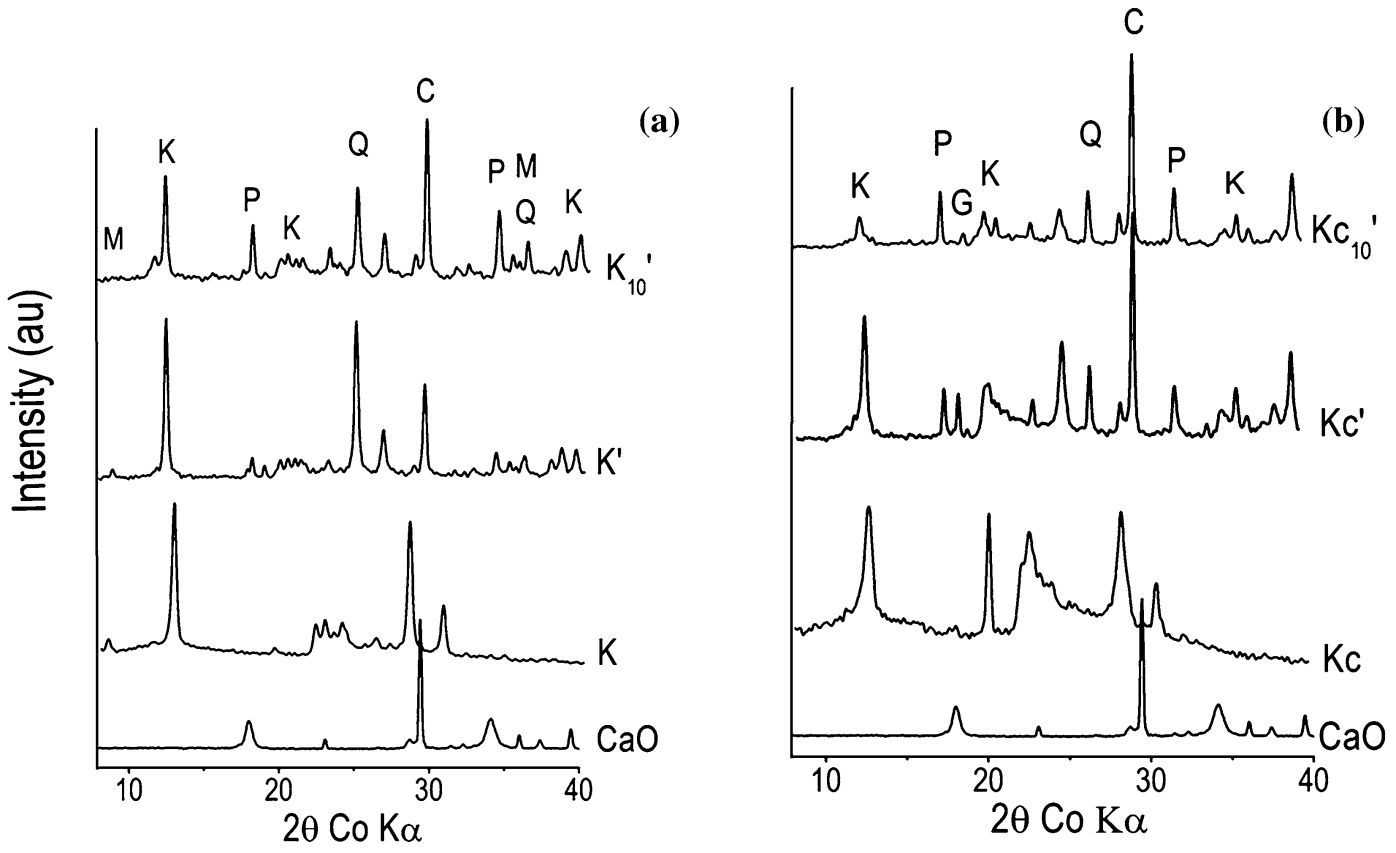
XRD patterns of materials before and after the Chapelle test: (**a**) CaO, K, K’ and K_10_′; (**b**) CaO, Kc, Kc’ and Kc_10_′ for which K: Kaolinite; Q: Quartz; C: calcite; M: Muscovite, G: Gibbsite and P: Portlandite.

The low amount of portlandite that remained after the reaction indicates the high pozzolanic activity of the studied sample. This is in agreement with Fig. 4 which presented the increase of pozzolanic activity of the two clays (K and Kc) with addition of 10 wt% of AH_3_ (from 535.0 to 724.7 mg of Ca(OH)_2_ /g in K and from 590.5 to 1,121.0 mg of Ca(OH)_2_ /g in Kc respectively), making the two clays fulfilling pozzolanic materials requirement according to NF P 18–513 Standard. As regard the improvement of pozzolanic activity of the two clays by addition of the same amount of 10 wt% of AH_3_, the increase is almost double in Kc, reaching 1,121.0 mg of Ca(OH)_2_/g, which is close to that of some Highly Reactive Metakaolins (1,500 mg of Ca(OH)_2_/g)^18^. Almost the same propensity is observed on the strength activity index of the clays alone and of those of mixtures of clays with aluminum hydroxide (Fig. 5). It can be seen that the strength activity index of the commercial clay K is lesser than that required for pozzolanic material, whereas that of Kc, which naturally contains gibbsite, is above the limit line. Finally, the addition of synthetic aluminum hydroxide (AH_3_) in the two clays led K into the sound zone (Fig. 4) and its activity index is even a bit greater than that of Kc according to ASTM C618 Standard^16,22^. This suggests the existence of a highest amount where aluminum hydroxide positively impacts the pozzolanic activity. Also, the strength activity index is a measurement of pozzolanic activity of materials aged 28 days, i.e. it measures the change of physical and mechanical properties in relation to the pozzolanic reaction. The decrease of the main peak intensity of gibbsite on XRD patterns of Chapelle test products (Fig. 6), which was evaluated at 90.8% from Kc to Kc’ and 96.5% from Kc to Kc_10_′, confirms that the initial gibbsite in Kc has also reacted with portlandite. This may be due to its possible low crystallinity as natural phase associated to kaolinite along with the amorphous aluminum hydroxide, which acted as an accelerator. Additionally, the Hinckley index (HI) of kaolinite in K’ evaluated at 1.6, seemed to have same value as in K_10_′, whereas HI increased from 0.6 in Kc’ to 1.9 in Kc_10_′ indicating that the addition of AH_3_ has no effect on the amount of amorphous phase in K. Conversely in Kc, it contributed considerably to consume the amorphous phase which led to the increase of the crystallinity of the kaolinite in Kc.

### Setting time of pastes

**Figure 7:**
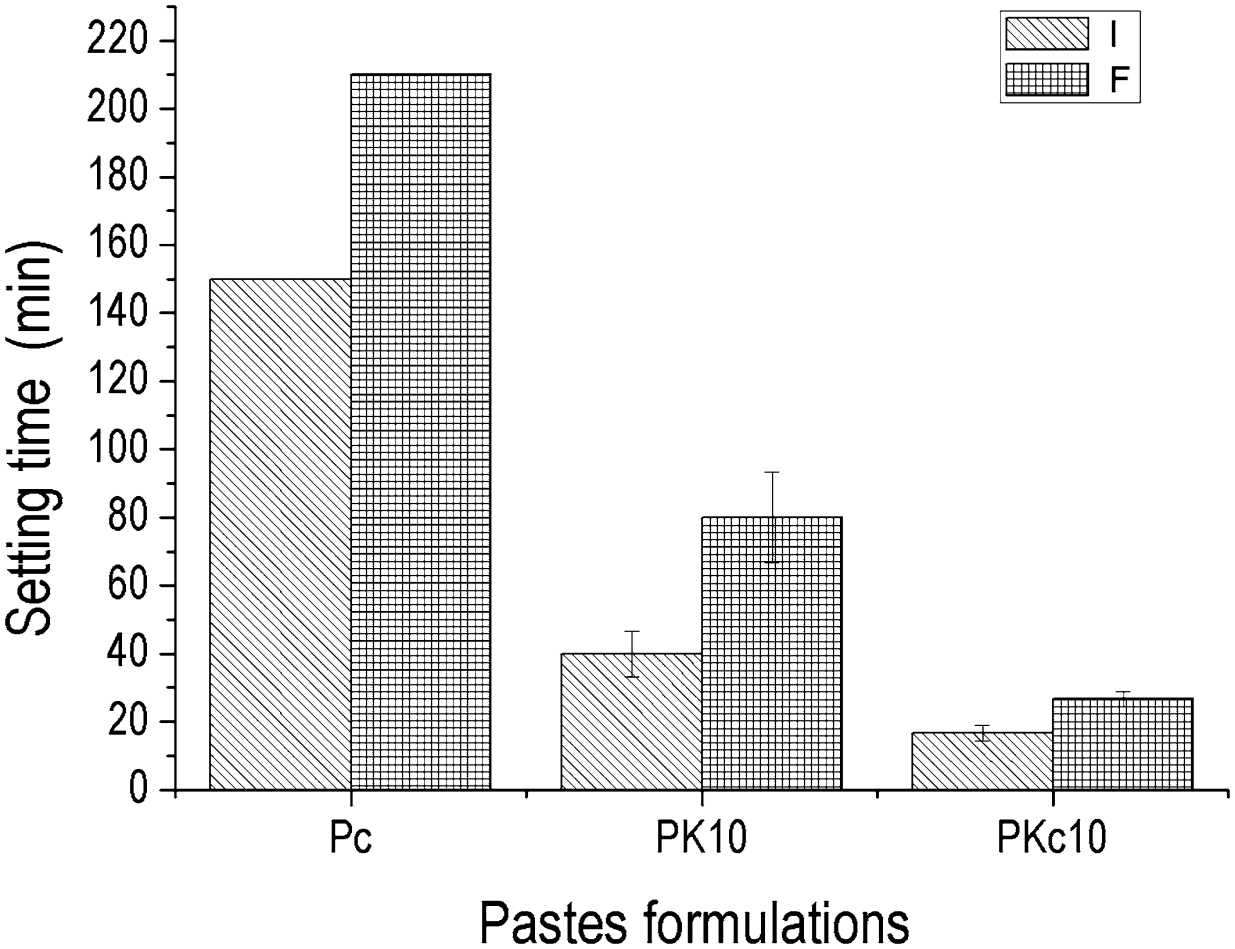
Setting time of different pastes, I: Initial setting time and F: Final setting time.

Figure 7 indicates the initial and final setting times of pastes which are respectively 150 and 210 min for control paste (PC), 40 and 80 min for PK_10_ and then 16 and 26 min in PKc_10_. It can be observed a rapid setting of the pastes with the addition of 10 wt% of amorphous aluminum hydroxide compared to the control paste. Once more, the role of amorphous aluminum hydroxide is highlighted as a material that generally affects the setting time^28^. It can also be stated that the amount of acting oxide (alumina) is a factor to be considered in the setting process. In fact, the amount of this oxide is greater in Kc due the presence of gibbsite and the effect is evident when considering the difference between setting time of PK_10_ and that of PKc_10_. This result could be connected to the value of the specific surface area (SSA) which is 51.0 m^2^/g for AH_3_, 17.0 m^2^/g for Kc and 13.5 m^2^/g for K, hence the higher the value of specific surface area, the lower the setting time. From the low values obtained for setting time, it is obvious that PKc_10_ and PK_10_ are suitable for manufacturing masonry blocks via extrusion or vibro-compaction process^29,30^.

### Mechanical strength of mortars

**Figure 8:**
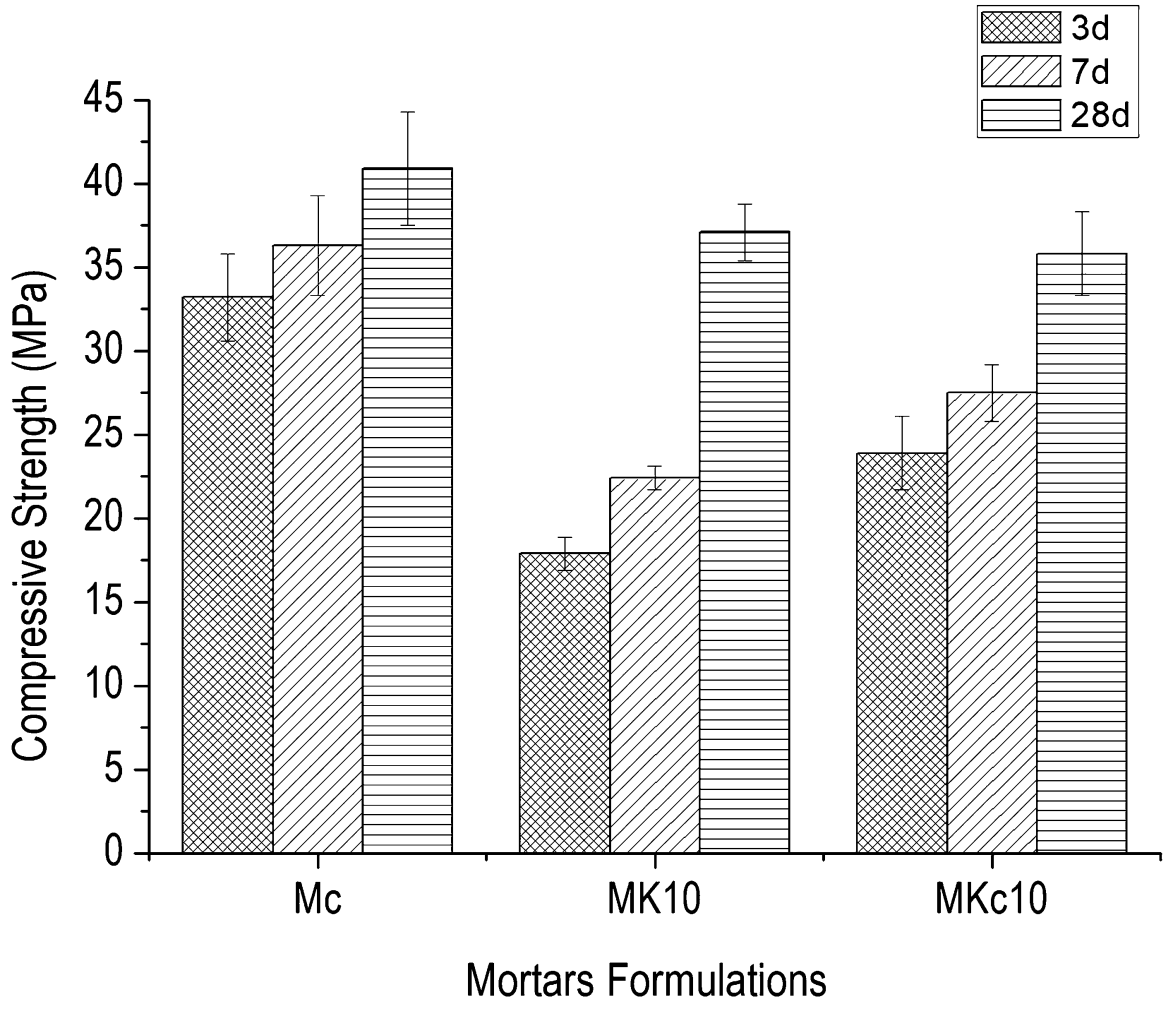
Compressive strength of mortars as function of age.

The variation of compressive strength with the age of the designed mortars (Mc, MK_10_ and MKc_10_) is shown in Fig. 8. The control mortar (Mc) specimens displayed highest values at all ages: 33.2 MPa (3 days), 36.3 MPa (7 days) and 41.0 MPa (28 days). Those of MK_10_ were 17.9 MPa (3 days), 22.4 MPa (7 days) and finally 37.1 MPa (28 days) whereas those of MKc_10_ were 23.9 MPa (3 days), 27.5 MPa (7 days) and finally 36 MPa (28 days). It can be observed that the compressive strength development was initially low at early ages and then significantly increased with time. It is well known that the hardening of the cement paste refers to the presence of hydrated phases. In the case of rapid setting time as observed in PK_10_ and PKc_10_ (Fig. 7), in addition to the formation of ettringite, the system undergoes instable and disorder structures of hydrated aluminosilcates (Eqs. 6–9) which is characterized by low mechanical strength as obtained for specimens aged 3 and 7 days^15,20,31,32^.

6 $$ 2{\text{Al}}\left( {{\text{OH}}} \right)_{3} \,(aluminum\,hydroxide) + 3{\text{Ca}}\left( {{\text{OH}}} \right)_{2} (portlandite) + 3\left( {{\text{CaSO}}_{4} \cdot 2{\text{H}}_{2} {\text{O}}} \right)\,(Gypse) + 20{\text{H}}_{2} {\text{O}}\,(water) \to 3{\text{CaO}} \cdot {\text{Al}}_{2} {\text{O}}_{3} \cdot 3{\text{CaSO}}_{4} \cdot 32{\text{H}}_{2} {\text{O}} \cdot (ettringite) $$

7 $$ \left( {{\text{CaO}},{\text{ Al}}_{2} {\text{O}}_{3} } \right) + {\text{H}}_{2} {\text{O}} \to {\text{CAH}}_{10} + {\text{C}}_{2} {\text{AH}}_{8} \,(Cement\,hydrates) $$

8 $$ {\text{CAH}}_{10} + {\text{Alumina}}\,{\text{gel}} \to {\text{C}}_{3} {\text{AH}}_{6} + {\text{AH}}_{3} $$

9 $$ {\text{C}}_{2} {\text{AH}}_{8} + {\text{Alumina}}\,{\text{gel}} \to {\text{C}}_{3} {\text{AH}}_{6} + {\text{AH}}_{3} $$

Furthermore, as the hydration proceeds, the initially formed metastable hydrated phases (CAH_10_ and C_2_AH_8_) are transformed into more stable ones such as C_3_AH_6_ with water (Eqs. 6–9). Thus, rapid setting time and formation of metastable phases due to the presence of amorphous aluminum hydroxide limited the cement grains from interacting with the surrounding water molecules. The impending access of water molecules to the surface of cement grains remained until the exhaustion of aluminum compound. This allowed the normal transformation mechanism of cement to more stable binder phases, which controlled the final mechanical strength. The aluminum compound also favoured the hardening, which attains a steady state at a limited amount^15,20,32^. Accordingly, in the formulation MK_10_, it can be suggested that the added aluminum hydroxide had, however, been involved in the process, therefore the contribution of gibbsite must also be considered. In accordance with this observation, the low values of compressive strength obtained at 3 and 7 days respectively in MK_10_ and MKc_10_ are contiguous to the presence of temporal phases as mentioned above and their presence can only be a source of insubstantial toughness or supplementary internal stress which favours low mechanical strength. Conversely, for specimens aged 28 days, the prevalence of more stable phases is effective and this leads to the higher values of mechanical strength. If the amount of amorphous aluminum hydroxide is a restrictive factor to the increase of mechanical strength, this is different with the pozzolanic activity which increases with the amount of aluminum compounds. Costa and Massazza^33^ showed that the pozzolanic reaction with lime depends on the specific surface area of the material at early ages. At later age, it depends more on the content of reactive compounds.

### Structural and microstructural characteristics of mortars

**Figure 9:**
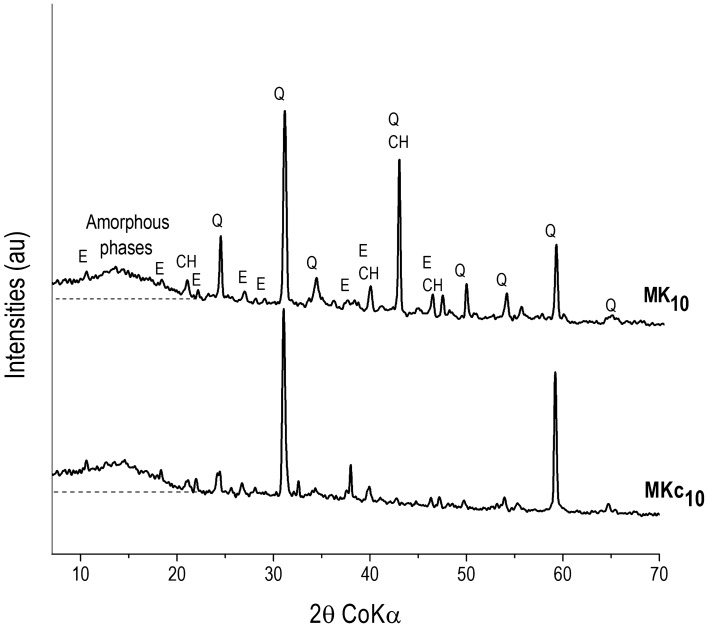
XRD patterns of MK10 and MKc10: CH (Portlandite), E (Ettringite), and Q (Quartz).

**Figure 10:**
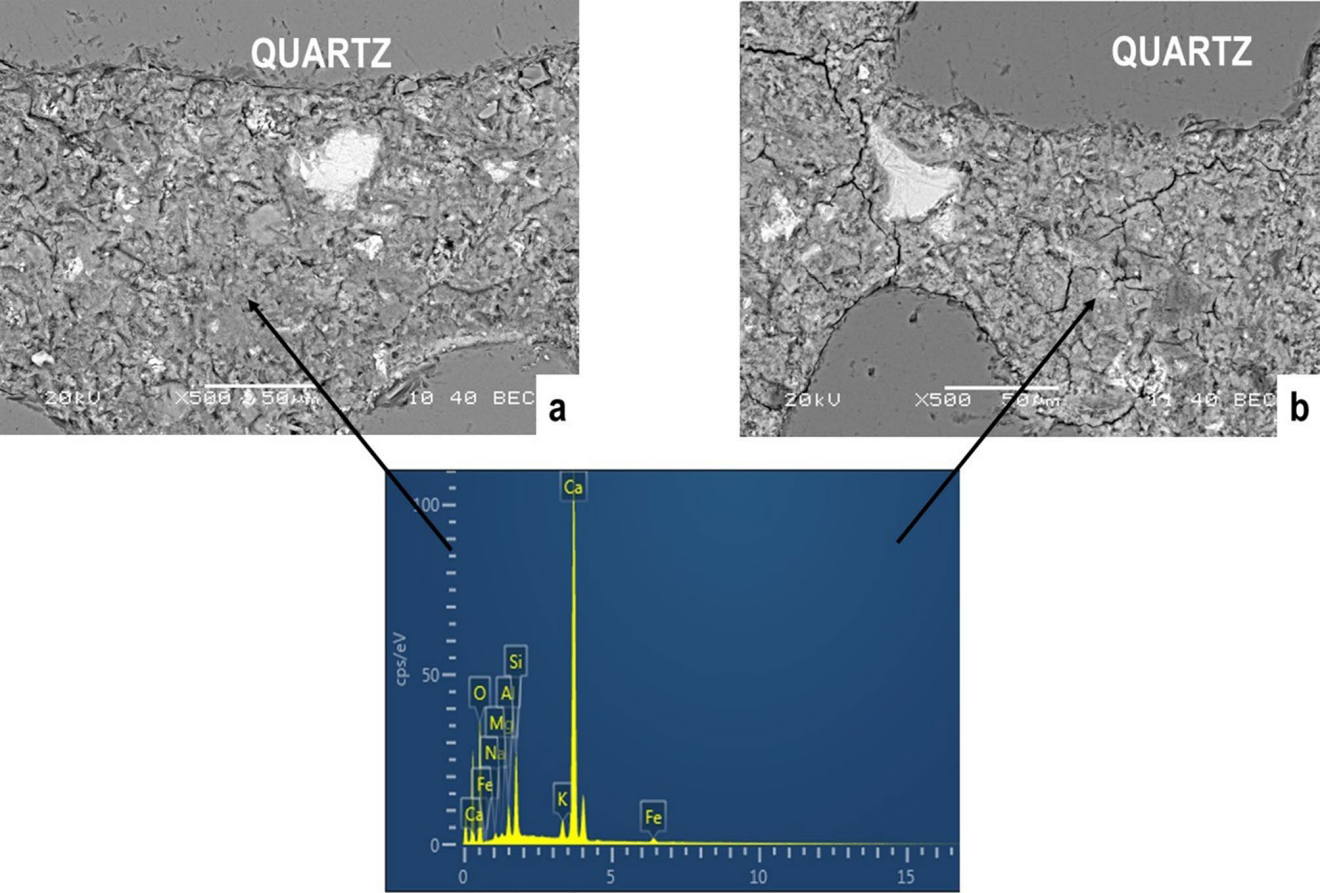
SEM images of polished cement mortar fracture of MK10 (**a**) and MKc10 (**b**) with EDX curve from the matrix zones.

XRD patterns of all mortars, MK_10_ and MKc_10_, (Fig. 9) show the presence of ettringite (C_6_AS_3_H_32_) as the main hydrated cement phase, and residual portlandite (Ca(OH)_2_) and quartz (SiO_2_) from sand. Considering that ettringite, a beneficial component of the cement system with an important role in the control of setting, is often formed preferably in the presence of alumina instead of its pair tobermorite whose formation is favored in presence of silica and high temperature process, the obtained result is therefore in accordance with the effective impact of amorphous aluminum hydroxide (AH_3_) in the mixture^25,26,34^. On the other hand, differences related to the intensity of peaks (Fig. 9) showed that their height is more or less high in accordance with the relative prevalence of respective concerned phases. The halo, which is observed at 10—20° (2θ), refers to the occurrence of amorphous phases^35,36^. As for the crystalline phases, slight dissimilarities can be seen with regard to the height of halo which is comparatively the same in MK_10_ and MKc_10_. In fact, the height of the halo is proportional to the amount of amorphous phases present in the system^37^. These amorphous phases are shown on SEM images (Fig. 10) and are specified by EDX curve from matrix zones as C–A–S–H and C–S–H^38,39^. It can then be concluded that the addition of 10 wt% of AH_3_ increased the formation of hydrated phases. All the SEM images of the mortars displayed a clear heterogeneous character. The background that was taken as matrix zone is constituted of the above mentioned amorphous phases. Within this matrix are erratically distributed large grains of sand and fine grains of crystalline hydrated phases which are identified on XRD patterns (Fig. 9). The overall microstructure highlights a compact tridimensional network where the matrix formed by amorphous phase gel is acting as a binder of aggregate grains along with other crystalline phases. In the network of the structure, the role of amorphous phases is mainly to control the strengthening of the mortars and their predominance is advantageous for mechanical strength of the mortars^23^. Accordingly, a slight relationship, even if not accurate at all, can be established between the obtained compressive strength of mortars and the height of amorphous halo (Figs. 8 and 9). Accordingly, the compressive strength of specimens aged 28 days is higher in MK_10_ (37.1 MPa) than in MKc_10_ (36.0 MPa).

## Conclusion

This paper addresses the use of clays in cement production in a context of saving natural resources and reducing CO_2_ emissions. The study investigated the use of commercial amorphous aluminum hydroxide in addition to raw kaolinite and the use of such mixtures as supplementary cementitious materials for partial replacement of cement in the formulation of cement mortars. The following conclusions were retained:

1. The incorporation of amorphous aluminum hydroxide favored the enhancement of pozzolanic activity of kaolinitic clays. The enhancement was greater when the clay originally contained gibbsite;
2. In comparison to the control paste made up of cement only, the presence of amorphous aluminum hydroxide in clays promoted significant decrease of initial and final setting times of cement pastes;
3. Amorphous aluminum hydroxide stimulated the formation of metastable hydrated phases by inhibiting temporally the hydration reactions of cement at the early age and with time, gradual transformation process led to the formation of more stable hydrated phases;
4. Raw kaolinitic clays containing aluminum hydroxide are promising substitute materials when blended with Portland cement. The sequence of transformation reactions with time is fully obtained with limited amorphous aluminum hydroxide content in clays. Even though, compressive strength is slightly reduced however the new cement meets the standard requirements for the construction sector.
5. The quick setting induced by amorphous aluminum hydroxide is an advantage for the elaboration of bricks or cement mortars using extrusion or vibro-compaction.

## Data Availability

The datasets analysed during the current study are available from the corresponding author on reasonable request.
